# In Monarchs, Cry2 Is King of the Clock

**DOI:** 10.1371/journal.pbio.0060012

**Published:** 2008-01-08

**Authors:** Richard Robinson

Internal clocks govern the daily rhythms of the most basic functions of waking, eating, and sleeping. The molecular basis of such pacemakers is best understood in fruit flies, where the interactions of a small set of molecules drive the insects' behaviors just as they do our own. The precise inner workings of each species' clock differ, however, as do the range of functions that clocks serve in each. In a new study, Haisun Zhu, Steven Reppert, and colleagues reveal the details of the clockwork in the monarch butterfly and show that it has aspects of clocks from both the mouse and the fly, the only other clock types known in animals. They further show that in addition to controlling the insect's daily activities, the butterfly clock may also aid its navigation during cross-continent migration.

In fruit flies, five proteins are central to the operation of the clock. Clock (Clk) and Cycle (Cyc) bind together to form a transcriptional complex, which drives production of Period (Per) and Timeless (Tim). Per and Tim then link up to repress the production of Clk and Cyc, thus creating a negative-feedback loop controlling the concentrations of Per and Tim—the essence of the clock mechanism. The fifth protein, Cryptochrome (Cry), is light sensitive, and when it absorbs blue light, it disrupts the Per–Tim complex, thereby resetting the periodic oscillations, which keeps the molecular clock and it output rhythms in tune with the ambient light-dark cycle. The mammalian clock uses Clk and a Cyc-like protein, but it has no Tim. Instead of a single cryptochrome, it has two, neither of which is light sensitive, but both of which are the major repressors of the mammalian clock feedback loop.

Previous work in the monarch has shown that it expresses its own version of Per, Tim, Clk, and Cyc, along with two cryptochromes: a fly-like cryptochrome, Cry1, and a mammalian-like cryptochrome, Cry2. The authors began their investigation into the detailed workings of the clock by showing that, as in the fly, the concentrations of Per and Tim fluctuate diurnally in the brains of the monarch. In a monarch cell line, they showed that while Cry1 absorbed light and triggered Tim degradation, the critical role in repressing Clk-Cyc transcription fell to Cry2. Since the mammalian orthologs of this protein play the same role in the mouse, it appears that Cry2 is the central timekeeper in two of the three known clocks (comparative genetic studies indicate Cry2 was lost in the *Drosophila* line).

**Figure:**
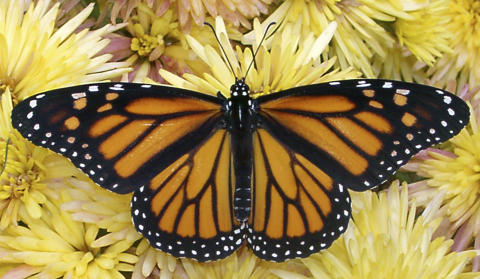
Two cryptochrome proteins, which act as critical components in an ancestral circadian clock mechanism characterized in monarch butterflies, may also connect the clock to the sun compass for successful navigation

Back in the brain, the authors showed that Cry2 was also found in a few dozen cells in brain regions previously linked to time-keeping in the butterfly, and this Cry2 underwent circadian oscillation in these cells, but not in many other cells that were not involved in time keeping. By taking samples periodically over many hours, they found that nuclear localization of Cry2 coincided with maximal transcriptional repression of the clockwork, in keeping with its central role of regulating the feedback cycle. This is a novel demonstration of nuclear translocation of a clock protein outside flies.

Finally, the authors investigated Cry2's activity in the central complex, the brain structure that is believed to house the navigational compass of the monarch. Monarchs integrate information on the position of the sun and the direction of polarized light to find their way from all over North America to the Mexican highlands, where they spend the winter. Cry2, but not the other clock proteins, was detected in parts of the central complex where it undergoes strong circadian cycling. Some cells containing Cry2 linked up with the clock cells, while others projected toward the optic lobe and elsewhere in the brain.

Along with highlighting the central importance of Cry2 in the inner workings of the monarch's clock, the results in this study suggest that part of the remarkable navigational ability of the butterfly relies on its ability to integrate temporal information from the clock with spatial information from its visual system. This allows the monarch to correct its course as light shifts across the sky over the course of the day. Other cues used for charting its path remain to be elucidated.

